# A longitudinal three-dimensional analysis of nasolabial morphology following primary cheiloplasty of unilateral cleft lip deformity

**DOI:** 10.3389/fdmed.2026.1783578

**Published:** 2026-04-17

**Authors:** Kei Yoshida, Kiyohide Ishihata, Motoko Tsuruda, Shinki Serizawa, Aiko Kurosaka, Namiko Kimura, Masahiro Tezuka, Toshiro Kibe, Tadashi Yamanishi, Hideto Saijo

**Affiliations:** 1 Department of Oral and Maxillofacial Surgery, Field of Oral and Maxillofacial Rehabilitation, Kagoshima University Graduate School of Medical and Dental Sciences, Kagoshima, Japan; 2 Department of Oral and Maxillofacial Surgery, Osaka Women’s and Children’s Hospital, Osaka, Japan

**Keywords:** cleft lip, Cronin method, longitudinal analysis, primary cheiloplasty, three-dimensional morphology

## Abstract

**Background:**

Primary cheiloplasty is the first definitive surgical intervention for infants with cleft lip and plays a central role in establishing the foundation of postoperative nasolabial morphology. Because the early configuration of the lip and nose can influence long-term facial harmony, achieving an anatomically favorable morphology at the time of initial surgery is an important clinical objective. At our institution, primary cheiloplasty is performed using the Cronin method in combination with nasal vestibular expansion (NVE), a technique designed to improve symmetry by simultaneously repositioning the soft tissues of both the lip and the nose. However, the extent and pattern of nasolabial morphological change during the early postoperative period and throughout the first year of growth remain insufficiently understood. This study therefore aims to longitudinally investigate postoperative changes in nasolabial morphology following primary cheiloplasty performed using the Cronin method with NVE.

**Methods:**

Twenty children with unilateral complete cleft lip were included in this study. Three-dimensional (3D) soft-tissue images were obtained using the Vectra H1® system at 1, 3, and 6 months and 1 year after surgery. An image analysis was performed using HBM-Rugle®, and symmetry was assessed by comparing the affected and non-affected sides. A reference plane was defined using the bilateral endocanthion points and the alar base point on the non-cleft side to ensure consistent spatial measurement of 3D coordinates.

**Results:**

The cleft-to-non-cleft side ratios of the alar base were 0.85–0.93 for the *X*-coordinate, 1.01–1.07 for the *Y*-coordinate, and 1.06–1.22 for the *Z*-coordinate across the four time points. Ratios for white-lip length were 0.86, 0.86, 0.84, and 1.02, whereas nasal-floor length ratios were 1.31, 1.38, 1.50, and 1.41. These trends indicated a slight deterioration in anteroposterior symmetry of the alar base and nasal floor over the first postoperative year, while white-lip length progressively improved and approached near-symmetry by 1 year.

**Conclusions:**

This longitudinal 3D analysis demonstrates region-specific postoperative changes within the nasolabial complex, suggesting that individual anatomical components exhibit distinct growth or relapse patterns. These findings may help refine surgical planning and early postoperative management aimed at optimizing nasolabial morphology following primary cheiloplasty with NVE.

## 1 Background

Primary cheiloplasty is recognized as the fundamental surgical intervention for patients with cleft lip and palate (CLP), serving as the first and most essential step in their multidisciplinary treatment (1–4). Beyond simply closing the cleft defect, this procedure is of paramount importance because it restores continuity of the upper lip, improves oral function, and establishes the foundation for the overall facial aesthetics of the patient (2, 5–7). Importantly, the outcomes of primary cheiloplasty have long-lasting implications, as the reconstructed tissues influence the nasolabial morphology throughout growth and development (1, 8, 9). Classic anthropometric and longitudinal studies have demonstrated that the nasolabial region continues to undergo differential growth after surgical repair, and that early surgical outcomes may be modified by growth-related changes and scar-related forces (10).

In our institution, we routinely employ the Cronin method in combination with nasal vestibular expansion (NVE). This surgical approach is designed to simultaneously correct lip and nasal deformities, with the goal of achieving a harmonious and well-balanced nasolabial morphology from the outset (11, 12). Nevertheless, despite the widespread adoption of primary cheiloplasty and numerous technical refinements reported in the literature, the long-term stability of nasolabial morphology remains an area of ongoing concern (1, 6, 8). Postoperative relapse, scar contracture, and the complex effects of craniofacial growth may alter the initially favorable outcomes (9). Previous studies have provided valuable cross-sectional observations; however, there is still a lack of comprehensive longitudinal evidence tracking how nasolabial morphology changes over time following primary cheiloplasty (5, 7, 13, 14). A deeper understanding of these changes is critical not only for refining surgical techniques but also for informing long-term treatment planning and counseling patients and their families about expected outcomes (2).

The present study was therefore designed to longitudinally evaluate patients who underwent primary cheiloplasty with the Cronin method combined with NVE. Our specific aim was to document progressive changes in nasolabial morphology across different growth stages, thereby clarifying the extent of relapse and growth-related modifications, and ultimately contributing to the establishment of more reliable treatment strategies for patients with cleft lip.

## 2 Materials and methods

### 2.1 Patients

Subjects in this study included 20 patients, 12 boys and eight girls, with complete cleft lip, who underwent primary lip repair in accordance with our treatment procedure between 2017 and 2024 (11, 15) (Table 1). This retrospective study included 20 patients with unilateral cleft lip for whom complete postoperative three-dimensional data were available at all scheduled time points (1, 3, 6 months, and 1 year after surgery). Only patients with a full set of follow-up records were included in the analysis. No cases were lost to follow-up during the 1-year postoperative period. Procedures were carried out by the same surgeon in the Department of Oral and Maxillofacial Surgery, Kagoshima University Medical and Dental Hospital. The treatment schedule included the following: (1) presurgical orthopedics using NAM from birth to primary lip repair, in order to minimize surgical intervention; (2) primary lip repair by the modified Cronin's triangular-flap method at 3–6 months old (mean age 5.1 ± 1.6 months) (12); (3) simultaneous medial upward advancement of nasolabial components, which allows repositioning of the lower lateral cartilage on the affected side; (4) vestibular expansion using a cleft margin approach to provide the vertical height of the nasal alar and nostrils and to minimize deformations due to postoperative regression; and (5) reconstruction of the orbicularis oris muscle, pars peripheralis, and pars marginalis, with overlapping, interdigitation, and edge-to-edge suturing, in order to reconstruct forms corresponding to the philtrum column (15, 16). Data were collected from the records of the CLP Team at Kagoshima University Medical and Dental Hospital. This study was approved by the Kagoshima University Medical and Dental Hospital Institutional Review Board (Approved No: 250063), and all participants provided informed consent.

**Table 1:** Characteristics of the subjects.

Sex	Male	12	Side of cleft	Right	5	Type of cleft	CL	3
	Female	8		Left	15		CLA	8
							CLP	9

*N* = 20. CL, lip only; CLA, lip + alveolus; CLP, lip + alveolus + palate.

### 2.2 Scanning device, image processing software, and data capture technique

The three-dimensional (3D) facial images of patients were captured using the non-invasive Vectra H1® 3D image scanner. The time points for image acquisition were 1, 3, and 6 months postoperatively, as well as 1 year after surgery. The face of the patients was captured from the front and diagonally downward at an angle of 45 on the right and left sides using the Vectra H1®. Each patient's facial image was captured several times, and images with similar expressions were selected and used. In order not to induce any differences in facial expression, the patients were placed in a calm environment and images were taken when their face was at rest.

### 2.3 Setting the reference plane

**Figure 1:**
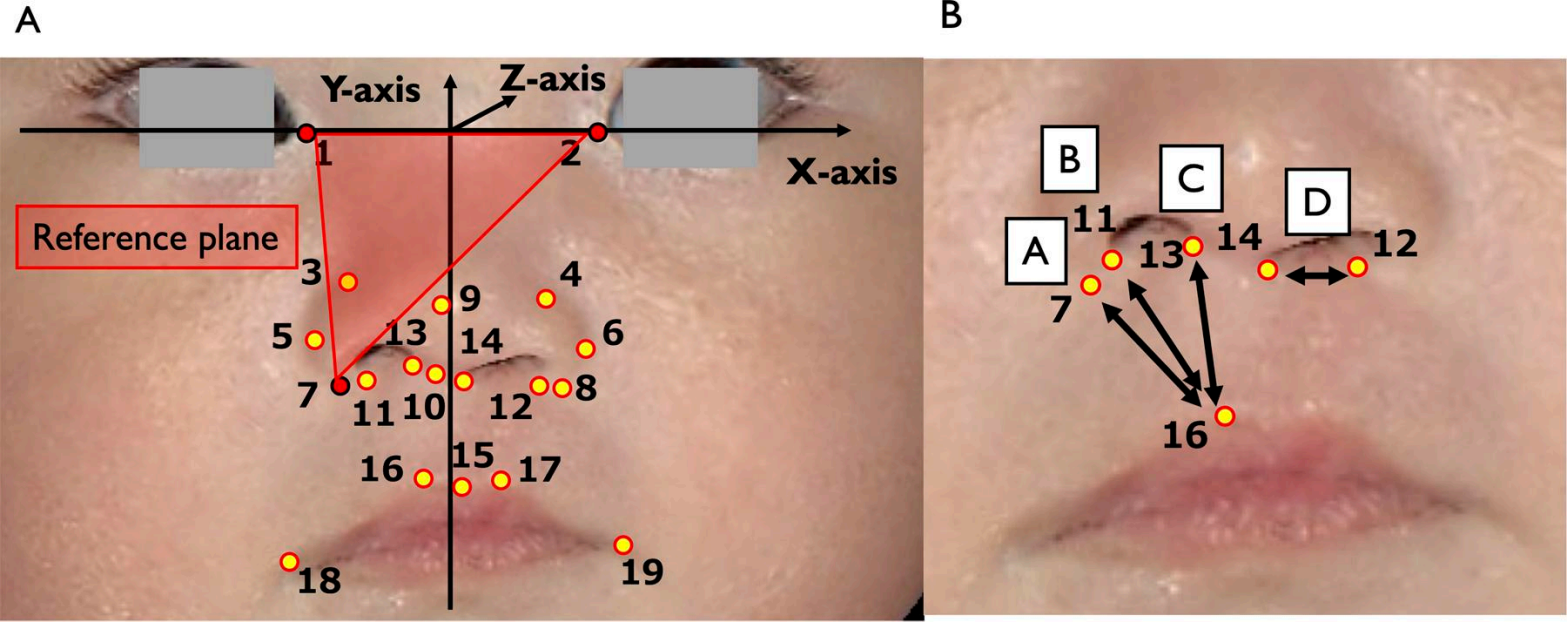
**(A)** The reference plane, axis, and facial landmarks 1–17 on a 3D facial texture image (front view). In order to determine the reference plane, the midpoint between the right and left endocanthion points was established as the origin (*x* = 0, *y* = 0, and *z* = 0). The plane containing the origin, left or right endocanthion, and the nasal alar base point of the non-cleft side was defined as the *x*-*y* plane (*z* = 0). **(B)** Measurement of white lip length and nasal base length. The white lip length was measured at three locations: (A) the distance from Alb (7, 8) to Lt (16, 17); (B) the distance from G.base (11, 12) to Lt (16, 17); and (C) the distance from Sbal (13, 14) to Lt (16, 17). The nasal base length was defined as (D) the distance between G.base (11, 12) and Sbal (13, 14).

In the next step, setting the reference planed measurements and analyses of nasolabial forms were performed using the 3D image software 3D—Rugle 7® (Medic Engineering, Inc., Kyoto, Japan). 3D—reconstructed facial images were compiled and standardized to face in the same direction, such that all clefts were presented on the left side. In each 3D facial image, the midpoint between the left and right endocanthion (En) points was set as the origin (point 0: *x* = 0, *y* = 0, and *z* = 0). The straight line passing through the left and right En was set as the *x-*axis, and the reference plane passing through three points, the right and left En and the nasal alar base point (Alb) on the non-cleft side, was set as the *x*–*y* plane. The straight line passing through the origin point and perpendicularly crossing the *x*-axis was set as the *y*-axis. Finally, the straight line passing through the origin point and crossing the *x*–*y* plane perpendicularly was set as the *z*-axis (Figure 1A). The reference plane was assessed three times separately for each 3D-reconstructed facial image. All procedures, including capturing, constructing, and analyzing 3D images, were performed by one examiner (NK) in order to avoid interexaminer errors (16).

### 2.4 Facial landmarks and measurement parameters

In order to assess nasolabial forms, 19 facial landmarks were detected on 3D facial images and placed manually onto texture images, while displaying normal vector images, so as to define facial structures more clearly (Table 2 and Figure 1A). Among these, 1–19 landmarks were found to match the anatomical locations used in previous studies of the nasolabial forms for patients with CLP (16–20). For each subject, the 3D coordinates of 19 facial landmarks were measured three times, and the mean value of each coordinate was used for analysis. At each measurement time point, the *x*-, *y*-, and *z*-coordinate values were compared between the cleft and the non-cleft sides. In addition, the white-lip length was evaluated at three locations, defined as the distances between Alb (points 7 and 8), G.base (points 11 and 12), Sbal (points 13 and 14), and Lt (points 16 and 17). The nasal floor length was measured as the distance between G.base (points 11 and 12) and Sbal (points 13 and 14) (Figure 1B). Subsequently, the ratio of the cleft and non-cleft sides was calculated.

**Table 2:** Facial landmarks.

No.	Abbreviation	Facial landmarks
1, 2	En	Endocanthion points of the non-cleft and cleft sides
3, 4	G. sup	Most superior points of the alar groove of the non-cleft and cleft sides
5, 6	G. lat	Most lateral points of the alar groove of the non-cleft and cleft sides
7, 8	Alb	Alar base points of the non-cleft and cleft sides; the intersection point of the straight line passing through the most lateral point on the alar groove of the non-cleft and cleft sides and the straight line passing through the columellar point.
9	Prn	Pronasale, nasal tip point; the highest point on the nasal tip
10	Col	Columellar base point; subnasal point
11, 12	G. base	Most inferior points of the alar groove of the non-cleft and cleft sides
13, 14	Sbal	Lateral point of the columellar base
15	Lm	Midpoint of Cupid's bow
16, 17	Lt	Top points of Cupid's bow of the non-cleft and cleft sides
18, 19	Ch	Cheilion points of the non-cleft and cleft sides; the point located at each labial commissure

### 2.5 Statistical analysis

Statistical analyses were performed using GraphPad Prism version 11 (GraphPad Software, San Diego, CA, USA). Normality of data distribution was assessed using the Shapiro–Wilk test prior to statistical comparisons. For normally distributed variables, paired Student's *t*-tests were used to compare measurements between the cleft and the non-cleft sides. For non-normally distributed data, the Wilcoxon signed-rank test was used. Longitudinal changes over time were analyzed using repeated-measures ANOVA for normally distributed variables and the Friedman test for non-parametric data. For landmarks 13–19, *x*-coordinate values were analyzed using absolute values to avoid directional effects and to focus on the magnitude of transverse asymmetry between sides.

Differences were considered statistically significant when *p* < 0.05. A *post hoc* power analysis was conducted based on the 1-month white-lip length (Length A) asymmetry data using a one-sample *t*-test against the symmetry value of 1.0. The observed effect size (Cohen's *d*) was 1.33. With a sample size of 20 and *α* = 0.05 (two-tailed), the calculated statistical power exceeded 0.99, indicating sufficient power to detect clinically relevant asymmetry. To assess measurement reliability, a randomly selected subset of cases was re-evaluated by the first and second authors. Each examiner performed all measurements three times at 2-week intervals. Intraexaminer reliability and interexaminer reliability were assessed using intraclass correlation coefficients (ICCs). The intraexaminer ICC values were 0.9973 and 0.9992 for the first and second authors, respectively, and the interexaminer ICC was 0.9976, indicating excellent reproducibility.

## 3 Results

### 3.1 Longitudinal changes in the *x*-, *y*-, and *z*-coordinates of external nasal morphology

**Figure 2:**
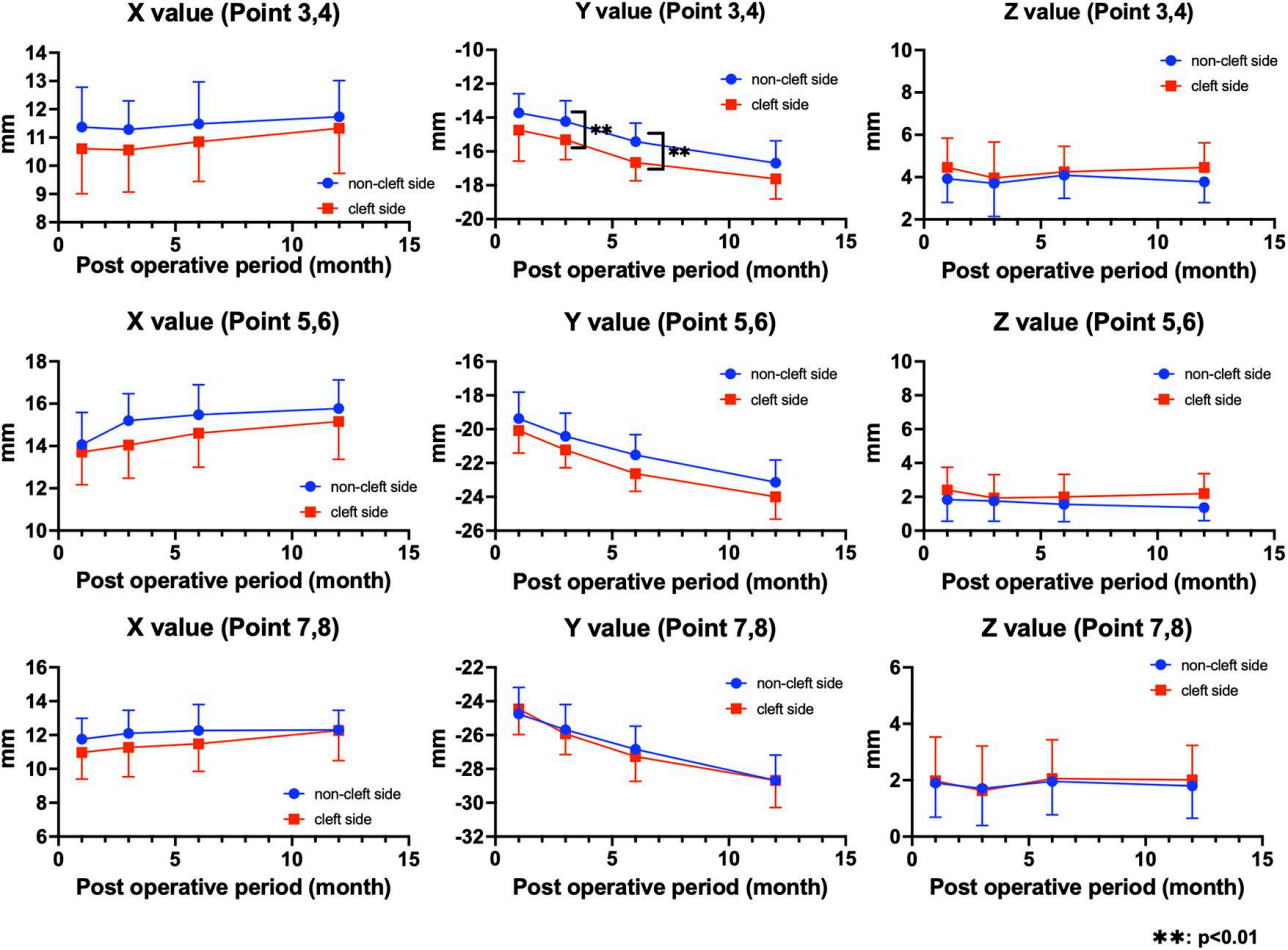
Longitudinal changes in the *x*-, *y*-, and *z*-coordinate values of external nasal landmarks on the cleft and non-cleft sides. The *x*-coordinate values at points 3–8 showed no statistically significant differences between the cleft and the non-cleft sides throughout the postoperative period, demonstrating a tendency toward reduced horizontal asymmetry. For the *y*-coordinate values, the cleft side at the superior alar rim (points 3 and 4) was positioned significantly lower at 3 and 6 months postoperatively (**: *p* < 0.01), and a difference remained at 1 year after surgery. The *z*-coordinate values indicated that, at 1 year postoperatively, the cleft side was located more anteriorly than the non-cleft side (points 4, 6, and 8).

With regard to external nasal morphology, no statistically significant differences were observed in the *x*-coordinate values, which ranged from 0.40 to 0.62 mm throughout the postoperative period, indicating a tendency toward reduced asymmetry between the cleft and the non-cleft sides. For the *y*-coordinate values at the superior alar rim point, the cleft side was significantly lower at 3 and 6 months postoperatively, and a difference of 1.1 mm persisted at 1 year after surgery. The *z*-coordinate values indicated that, at 1 year postoperatively, the cleft side was positioned more anteriorly (point 4: 0.6 mm; point 6: 0.8 mm; point 8: 0.58 mm) compared with the non-cleft side (Table 3; Figure 2).

**Table 3:** Longitudinal changes in three-dimensional coordinate values.

	Coordinate	Time point (month)	Non-cleft side (mm)		Cleft side (mm)		*p*-Value
			Mean	SD	Mean	SD	
Point 3, 4	*X*	1	11.37	1.41	10.60	1.59	0.201
		3	11.28	1.01	10.56	1.49	0.249
		6	11.48	1.49	10.85	1.40	0.368
		12	11.73	1.28	11.33	1.60	0.557
	*Y*	1	−13.72	1.12	−14.74	1.83	0.061
		3	−14.24	1.23	−15.31	1.17	**<0** **.** **01**
		6	−15.42	1.08	−16.66	1.08	**<0** **.** **01**
		12	−16.69	1.31	−17.62	1.18	0.309
	*Z*	1	3.93	1.12	4.46	1.39	0.462
		3	3.70	1.56	3.96	1.69	0.876
		6	4.09	1.10	4.25	1.21	0.051
		12	3.78	0.98	4.45	1.17	0.061
Point 5, 6	*X*	1	14.06	1.52	13.71	1.54	0.063
		3	15.20	1.27	14.05	1.57	0.102
		6	15.48	1.42	14.61	1.61	0.154
		12	15.77	1.35	15.16	1.79	0.375
	*Y*	1	−19.37	1.56	−20.08	1.33	0.228
		3	−20.42	1.37	−21.22	1.05	0.054
		6	−21.51	1.19	−22.63	1.05	0.062
		12	−23.14	1.32	−23.99	1.32	0.060
	*Z*	1	1.84	1.28	2.41	1.34	0.104
		3	1.75	1.19	1.94	1.37	0.622
		6	1.56	1.03	2.00	1.34	0.182
		12	1.36	0.76	2.19	1.18	0.056
Point 7, 8	*X*	1	11.76	1.23	10.98	1.58	0.147
		3	12.10	1.37	11.27	1.73	0.424
		6	12.27	1.54	11.49	1.63	0.264
		12	12.31	1.16	12.27	1.78	0.971
	*Y*	1	−24.76	1.57	−24.45	1.53	0.416
		3	−25.69	1.49	−25.93	1.23	0.537
		6	−26.84	1.38	−27.27	1.46	0.342
		12	−28.68	1.51	−28.68	1.60	0.803
	*Z*	1	1.90	1.21	1.98	1.55	0.966
		3	1.71	1.32	1.63	1.59	0.648
		6	1.96	1.19	2.06	1.38	0.831
		12	1.80	1.15	2.01	1.22	0.058

Bold values indicate variables with statistically significant differences (*p* < 0.05).

### 3.2 Longitudinal changes in the *x*-, *y*-, and *z*-coordinate values of landmarks around the Cupid's bow and columellar base on the cleft and non-cleft sides

**Figure 3:**
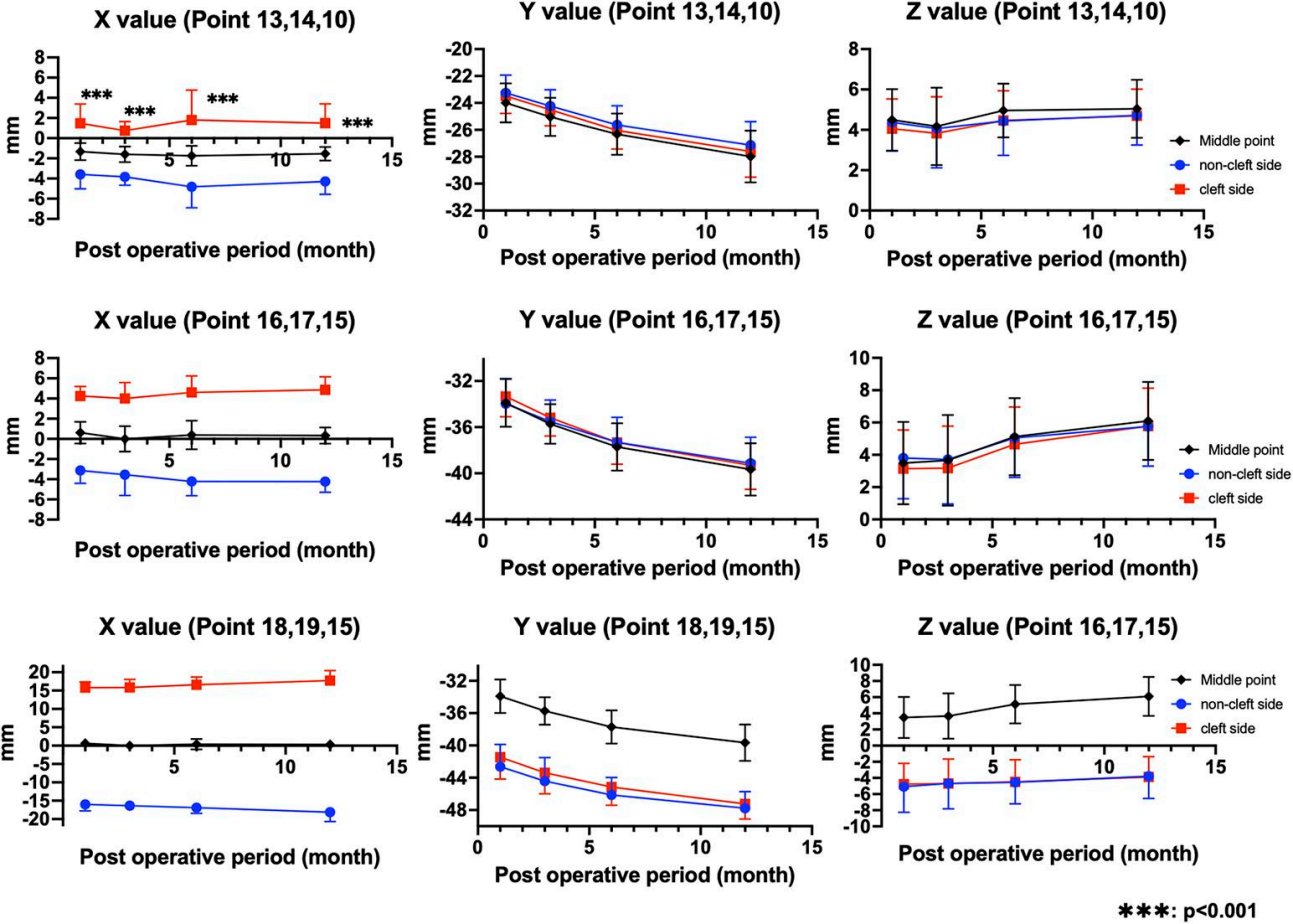
Longitudinal changes in the *x*-, *y*-, and *z*-coordinate values of landmarks around the Cupid's bow and columellar base on the cleft and non-cleft sides. The *x*-coordinate values at the columellar base demonstrated a significant horizontal asymmetry that persisted up to 1 year postoperatively, indicating incomplete resolution of transverse deviation despite early surgical correction (**p < 0.001). At the Cupid's bow, the interside difference in *x*-coordinate values progressively decreased during the postoperative period, suggesting a gradual improvement in horizontal symmetry. In contrast, the differences in the *y*- and *z*-coordinate values remained relatively stable throughout follow-up, reflecting limited postoperative change in the vertical position and anterior–posterior projection. Overall, postoperative remodeling contributed primarily to the reduction in horizontal asymmetry at the Cupid's bow, whereas residual positional discrepancies at the columellar base persisted during the first postoperative year.

A significant horizontal asymmetry at the columellar base was observed and persisted up to 1 year postoperatively, indicating incomplete resolution of transverse deviation despite early surgical correction. With respect to the Cupid's bow, the interside difference in *x*-coordinate values progressively decreased during the postoperative period (from 1.11 to 0.62 mm), suggesting a gradual improvement in horizontal symmetry. In contrast, differences in the *y*- and *z*-coordinate values remained relatively stable throughout follow-up (approximately 0.40–0.50 mm), indicating limited change in vertical position and anterior–posterior projection. Overall, these findings demonstrate that postoperative remodeling contributed primarily to reduction in horizontal asymmetry at the Cupid's bow, whereas residual positional discrepancies at the columellar base persisted during the first postoperative year (Table 4; Figure 3).

**Table 4:** Longitudinal changes in three-dimensional coordinate values.

	Coordinate	Time point (month)	Non-cleft side (mm)		Cleft side (mm)		*p*-Value	Middle point (mm)	
			Mean	SD	Mean	SD		Mean	SD
Point 13, 14, 10	*X*	1	−3.59	1.44	1.47	1.92	**<0** **.** **001**	−1.32	0.85
		3	−3.83	0.83	0.74	0.91	**<0** **.** **001**	−1.59	0.78
		6	−4.81	2.09	1.81	2.96	**<0** **.** **001**	−1.74	0.99
		12	−4.29	1.27	1.49	1.90	**<0** **.** **001**	−1.54	0.67
	*Y*	1	−23.26	1.32	−23.48	1.30	0.590	−24.00	1.45
		3	−24.23	1.21	−24.51	1.19	0.515	−25.03	1.42
		6	−25.63	1.43	−26.04	1.39	0.331	−26.33	1.54
		12	−27.14	1.75	−27.61	1.91	0.430	−27.99	1.93
	*Z*	1	4.37	1.42	4.06	1.47	0.510	4.49	1.52
		3	4.06	1.94	3.83	1.81	0.618	4.17	1.92
		6	4.43	1.70	4.46	1.48	0.736	4.96	1.33
		12	4.73	1.48	4.70	1.32	0.955	5.04	1.44
Point 16, 17, 15	*X*	1	−3.13	1.27	4.24	0.95	0.052	0.61	1.07
		3	−3.54	2.07	4.00	1.57	0.179	0.00	1.27
		6	−4.21	1.42	4.60	1.64	0.413	0.38	1.42
		12	−4.23	1.06	4.86	1.29	0.139	0.33	0.81
	*Y*	1	−33.98	2.18	−33.33	1.77	0.266	−33.90	2.07
		3	−35.51	1.86	−35.19	1.57	0.614	−35.72	1.71
		6	−37.31	2.16	−37.33	1.89	0.997	−37.71	2.05
		12	−39.13	2.26	−39.31	2.08	0.807	−39.67	2.27
	*Z*	1	3.80	2.52	3.14	2.39	0.309	3.49	2.55
		3	3.72	2.75	3.17	2.60	0.338	3.66	2.81
		6	5.04	2.43	4.65	2.30	0.569	5.12	2.38
		12	5.76	2.46	5.78	2.36	0.913	6.10	2.42
Point 18, 19, 15	*X*	1	−15.99	1.79	15.80	1.59	0.761	0.61	1.07
		3	−16.34	1.21	15.86	2.19	0.938	0.00	1.27
		6	−16.89	1.55	16.59	2.15	0.431	0.38	1.42
		12	−18.11	2.57	17.74	2.72	0.635	0.33	0.81
	*Y*	1	−42.62	2.76	−41.45	2.71	0.197	−33.90	2.07
		3	−44.42	2.91	−43.38	2.62	0.254	−35.72	1.71
		6	−46.13	2.16	−45.15	2.25	0.133	−37.71	2.05
		12	−47.78	2.07	−47.24	1.88	0.319	−39.67	2.27
	*Z*	1	−5.08	3.18	−4.76	2.56	0.778	3.49	2.55
		3	−4.68	3.15	−4.69	3.03	0.941	3.66	2.81
		6	−4.54	2.66	−4.49	2.73	0.930	5.12	2.38
		12	−3.79	2.76	−3.88	2.53	0.908	6.10	2.42

Bold values indicate variables with statistically significant differences (*p* < 0.05).

### 3.3 Longitudinal changes in white-lip length, nasal floor length, and cleft-to-non-cleft side ratios

**Figure 4:**
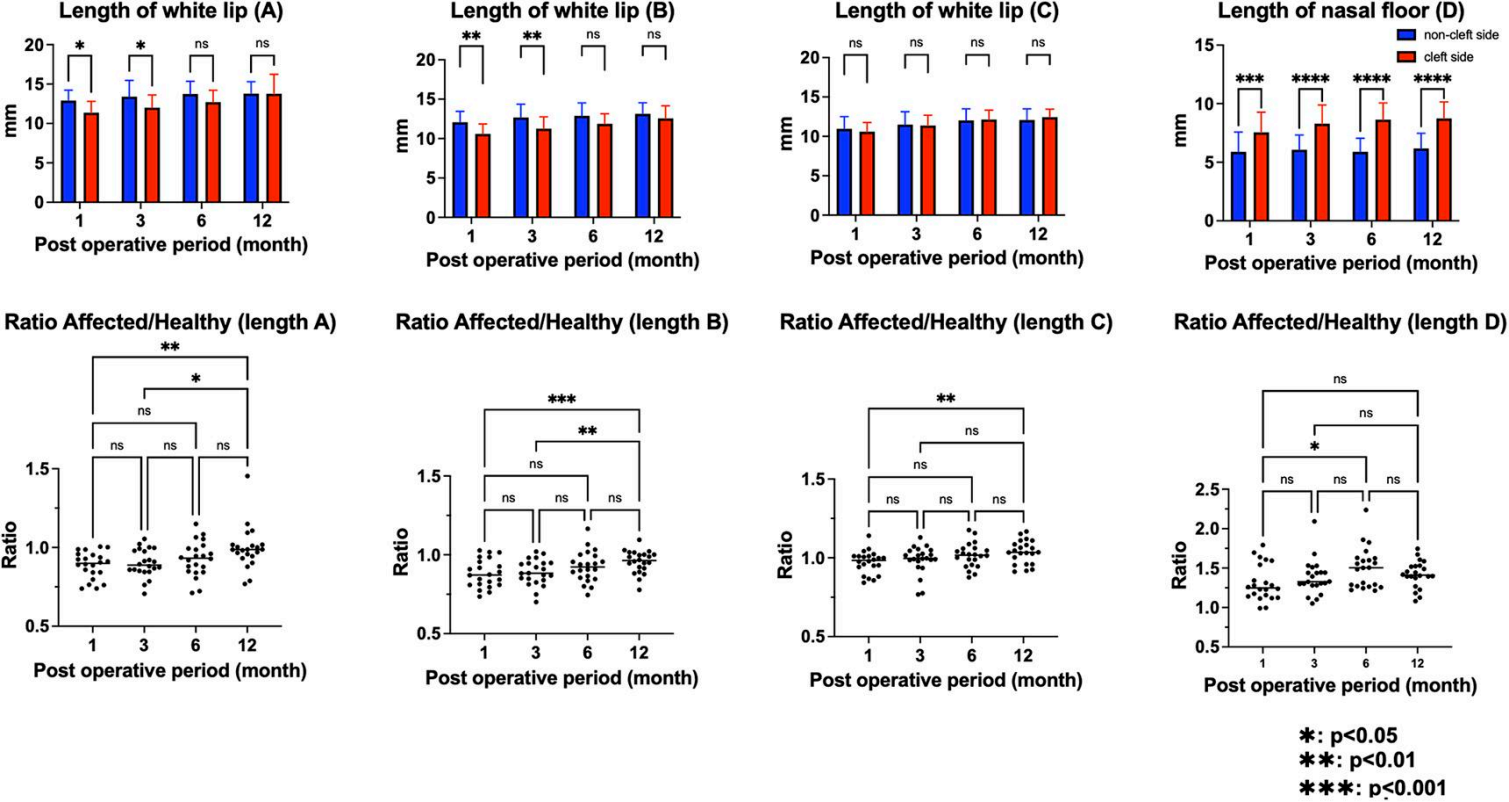
Longitudinal changes in white-lip length, nasal floor length, and cleft-to-non-cleft side ratios. The lateral white-lip length on the cleft side (lengths A and B) was significantly shorter than that on the non-cleft side during the early postoperative period (up to 1 and 3 months, respectively); however, these interside differences progressively diminished thereafter, and no significant differences were observed at 6 months and 1 year postoperatively. By contrast, the nasal floor length (length D) remained consistently greater on the cleft side throughout the entire observation period. To quantify the magnitude of asymmetry, cleft-to-non-cleft side ratios were calculated for each measurement at all time points (1, 3, and 6 months and 1 year): length A, 0.88, 0.89, 0.92, and 1.00; length B, 0.88, 0.88, 0.92, and 0.97; length C, 0.97, 0.98, 1.01, and 1.03; and nasal floor length (length D), 1.31, 1.38, 1.50, and 1.41, respectively. The asterisks indicate statistically significant differences between sides (*p < 0.05; **p < 0.01; ***p < 0.001); ns, not significant.

The cleft-side lateral white lip (lengths A and B) was significantly shorter than that on the non-cleft side during the early postoperative period (up to 1 and 3 months, respectively); however, these interside differences progressively diminished thereafter, and no significant difference was observed at 6 months and 1 year. By contrast, the nasal floor length (length D) remained consistently greater on the cleft side throughout the entire observation period. To further characterize the magnitude of asymmetry, cleft-to-non-cleft side ratios were calculated at each time point. The postoperative ratios at 1, 3, and 6 months and 1 year were as follows: length A, 0.88, 0.89, 0.92, and 1.00; length B, 0.88, 0.88, 0.92, and 0.97; length C, 0.97, 0.98, 1.01, and 1.03; and nasal floor length (length D), 1.31, 1.38, 1.50, and 1.41, respectively (Tables 5 and 6; Figure 4).

**Table 5:** Length of white lip and nasal floor.

Area	Time point (month)	Non-cleft side (mm)		Cleft side (mm)		*p*-Value
		Mean	SD	Mean	SD	
A	1	12.92	1.28	11.38	1.40	**0** **.** **015**
	3	13.41	2.01	12.01	1.58	**0** **.** **032**
	6	13.74	1.57	12.70	1.47	0.188
	12	13.80	1.47	13.77	2.42	0.987
B	1	12.07	1.35	10.59	1.24	**0** **.** **004**
	3	12.67	1.65	11.26	1.45	**0** **.** **008**
	6	12.89	1.59	11.87	1.26	0.094
	12	13.15	1.36	12.55	1.58	0.735
C	1	10.96	1.51	10.58	1.14	1.000
	3	11.49	1.60	11.37	1.30	1.000
	6	12.02	1.43	12.14	1.16	1.000
	12	12.07	1.38	12.44	1.00	1.000
D	1	5.90	1.65	7.55	1.69	**0** **.** **001**
	3	6.07	1.23	8.29	1.56	**<0** **.** **0001**
	6	5.89	1.12	8.64	1.39	**<0** **.** **0001**
	12	6.19	1.26	8.74	1.38	**<0** **.** **0001**

Bold values indicate variables with statistically significant differences (*p* < 0.05).

**Table 6:** Ratio non-cleft side/cleft side.

Area	Time point (month)	Non-cleft/cleft side	Dunn's multiple comparisons test, *p*-value				
		Mean	SD	1 month	3 months	6 months	12 months
A	1	0.89	0.08	-	1.000	0.613	**0** **.** **002**
	3	0.90	0.09	1.000	-	1.000	**0** **.** **030**
	6	0.93	0.11	0.613	1.000	-	0.283
	12	1.00	0.13	**0** **.** **002**	**0** **.** **030**	0.283	-
B	1	0.88	0.09	-	1.000	0.479	**<0** **.** **001**
	3	0.89	0.08	1.000	-	1.000	**0** **.** **004**
	6	0.93	0.10	0.479	1.000	-	0.117
	12	0.96	0.07	**<0** **.** **001**	**0** **.** **004**	0.117	-
C	1	0.97	0.07	-	0.213	0.061	**0** **.** **001**
	3	0.99	0.09	0.213	-	1.000	0.613
	6	1.01	0.08	0.061	1.000	-	1.000
	12	1.04	0.07	**0** **.** **001**	0.613	1.000	-
D	1	1.31	0.23	-	1.000	0.030	0.613
	3	1.38	0.22	1.000	-	0.213	1.000
	6	1.50	0.25	0.030	0.213	-	1.000
	12	1.42	0.16	0.613	1.000	1.000	-

Bold values indicate variables with statistically significant differences (p < 0.05).

No statistically significant differences were found between male and female patients at any postoperative time point (*p* > 0.05). Similarly, no significant differences were observed between right- and left-sided clefts after image standardization (*p* > 0.05).

## 4 Discussion

This study conducted a 3D longitudinal evaluation of nasolabial morphology after primary cheiloplasty using the Cronin method combined with NVE. Surgical outcomes in growing patients are influenced by both relapse related to scar contracture and alterations associated with craniofacial growth, and it is often difficult to clearly distinguish the contribution of each factor in the postoperative course. Nevertheless, understanding the direction and magnitude of these postoperative morphological changes is essential for determining the most appropriate immediate postoperative configuration and for optimizing long-term aesthetic and functional outcomes in patients with cleft lip (21, 22).

The present study revealed that postoperative remodeling did not occur uniformly across the nasolabial complex, but instead followed a region-specific pattern. Horizontal asymmetry at the Cupid's bow gradually improved over the first postoperative year, whereas residual asymmetry persisted at the columellar base. This observation suggests that the soft tissues in the philtral and lip region possess a greater capacity for postoperative adaptation than those around the nasal base; the latter may be more vulnerable to relapse or growth-related divergence and, in addition, continues to represent a region in which it is difficult to achieve complete correction during primary cleft lip surgery. The persistence of columellar base asymmetry is consistent with previous reports indicating that nasal base morphology is strongly influenced by scar tethering, cartilage deformation, and soft-tissue tension along the cleft margin (23, 24). In contrast, the nasal floor length remained consistently greater on the cleft side at all time points. Because the nasal floor plays a key role in defining alar width, nostril contour, and nasal base projection, even minor discrepancies may affect nasolabial harmony and facial symmetry (25). Previous anthropometric and photographic studies have similarly reported residual asymmetry of the nasal base after primary lip repair, although most of these investigations were cross-sectional rather than longitudinal in design (26, 27).

**Figure 5:**
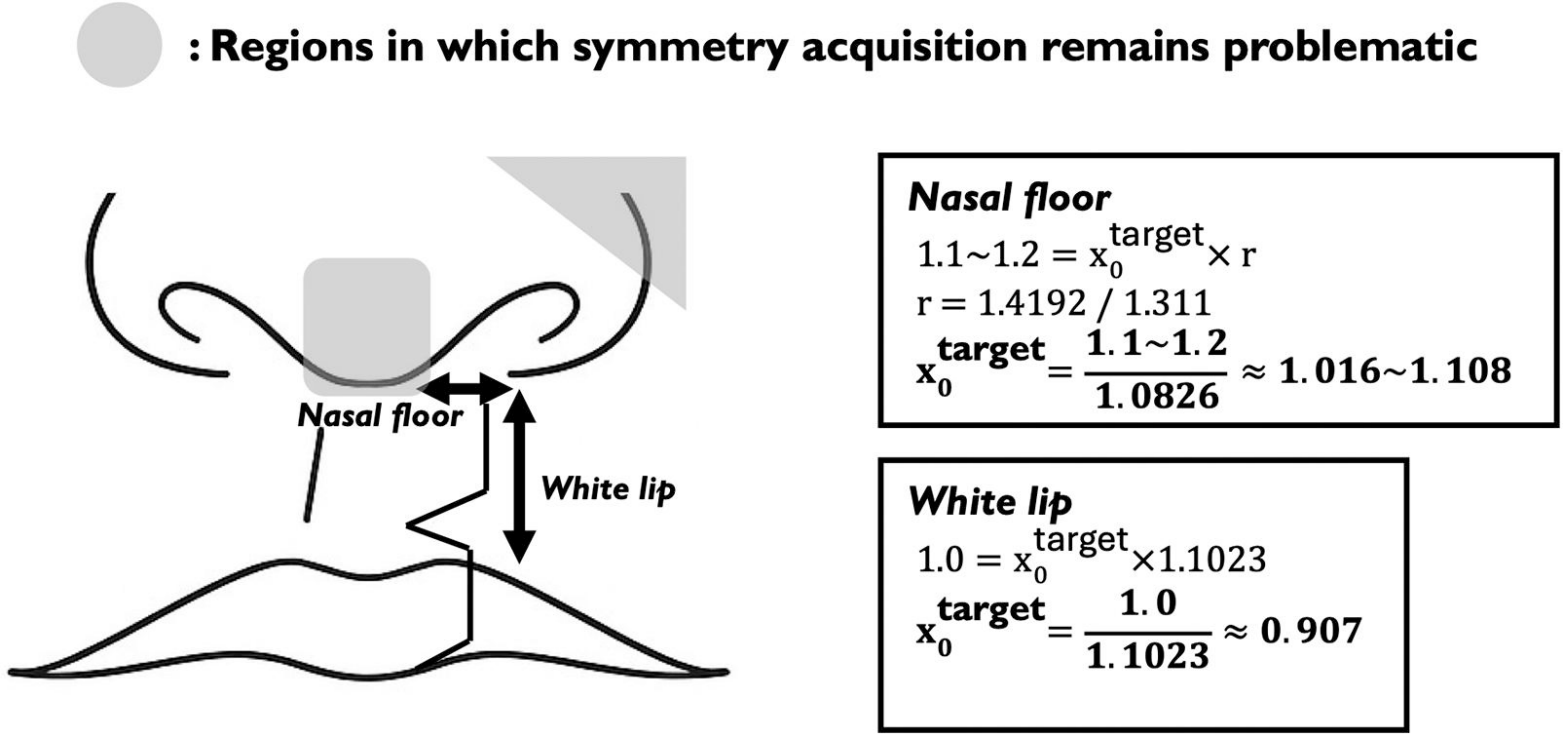
Recommended target ratios for cleft-to-non-cleft side measurements at primary repair. The diagram illustrates the recommended construction ratios at the time of primary cleft lip repair to facilitate symmetry at 1 year postoperatively. The gray shaded regions indicate areas where symmetry acquisition remains problematic and further improvement may be required. The white lip should be constructed at a cleft-to-non-cleft ratio of 0.907, while the nasal floor length should be intentionally shaped within the range of 1.016–1.108 in anticipation of secondary rhinoplasty in adulthood.

Through serial 3D assessments, this study demonstrated that the greater nasal floor width on the cleft side tended to be maintained during the first postoperative year, with little evidence of spontaneous improvement. Given that enlargement of a constricted nostril during secondary rhinoplasty in adulthood is technically demanding, these results offer objective evidence reinforcing the clinical rationale for careful construction of the nasal floor morphology at the primary cleft lip repair. That is, as illustrated in Figure 5, although the regions highlighted in gray indicate areas where further improvement may still be required, we would recommend, in order to facilitate symmetry at 1 year postoperatively, that the white lip length be constructed at a cleft-to-non-cleft ratio of 0.907 at the time of primary cleft lip repair, and that the nasal floor length be intentionally shaped within the range of 1.016–1.108 in anticipation of secondary rhinoplasty in adulthood (Figure 5).

The Cronin method, a modification of the triangular flap technique, is intended to reconstruct the philtral column while preserving adequate lip height. However, the additional open-cut incision may predispose the central lip to scar contracture, whereas postoperative elongation of the lateral lip has been described in prior clinical observations (12, 28). The present longitudinal findings—showing early postoperative shortening of the cleft-side lateral white lip followed by gradual recovery toward near-symmetry—may reflect the combined effects of scar maturation, soft-tissue accommodation, and reorganization of the reconstructed orbicularis oris muscle (29, 30). These adaptive changes contrast with the more static behavior of the columellar base and nasal floor, highlighting anatomical differences in tissue elasticity and mechanical constraint between regions. Recent advances in 3D imaging have enabled a more comprehensive analysis of surface morphology and symmetry, particularly in the early postoperative growth period (31, 32). The present study extends these findings by demonstrating that different elements of the nasolabial region follow distinct postoperative trajectories, rather than changing uniformly as a single functional unit. This perspective may help explain why aesthetic outcomes can vary even when an apparently favorable immediate postoperative morphology is achieved.

From a surgical planning standpoint, these results carry several important clinical implications. First, the progressive improvement in horizontal symmetry around the Cupid's bow suggests that intentional overcorrection in this region may not be necessary and could potentially increase the risk of secondary deformity during growth. Conversely, the persistent asymmetry of the nasal floor and columellar base indicates that greater emphasis should be placed on achieving adequate projection and transverse balance in these structures during primary repair, particularly when using techniques that may induce scar tightening along the cleft margin. Moreover, the present findings may provide useful information for parental counseling by clarifying which features are likely to remodel spontaneously and which may require secondary revision procedures at a later developmental stage.

This study also presents several limitations. First, the absence of a control group and preoperative baseline three-dimensional data limits the ability to distinguish surgical effects from early postoperative tissue remodeling and natural growth-related changes. Without comparison with alternative surgical techniques, historical growth data, or preoperative 3D measurements, the findings should be interpreted as descriptive longitudinal observations within a standardized treatment framework rather than definitive evidence of technique-specific surgical outcomes. In addition, all patients underwent presurgical NAM according to the same institutional protocol prior to surgery. Although NAM was uniformly applied across the cohort, its potential contribution to early postoperative morphology cannot be entirely ignored. Therefore, the observed changes may reflect the combined influence of presurgical orthopedic intervention and surgical repair. Second, the observation period was limited to the first postoperative year, and therefore, the influence of later craniofacial growth, dentoalveolar development, and subsequent surgical interventions could not be assessed. Previous longitudinal studies have shown that nasolabial morphology continues to change throughout childhood and adolescence (33, 34); thus, an extended follow-up is necessary to determine whether the asymmetries identified here persist, stabilize, or progress over time. Third, all procedures were performed using a single surgical method by one surgeon, which ensured methodological consistency but may limit generalizability to other cheiloplasty techniques. The present study included patients with unilateral cleft lip with or without alveolar and palatal involvement. Because the objective was to analyze longitudinal postoperative remodeling following a standardized surgical technique rather than to compare cleft subtypes, subgroup-specific analyses were not performed. However, anatomical heterogeneity may influence growth patterns, and future studies with larger homogeneous cohorts are warranted. Because this study was not designed or powered to detect differences among cleft subtypes, intergroup comparisons were not performed. Future investigations with larger homogeneous cohorts are warranted to clarify subtype-specific remodeling patterns. Comparative analyses across surgical approaches—including those with differing philosophies of muscle reconstruction and nasal cartilage repositioning—would further clarify technique-dependent patterns of postoperative change. Finally, although 3D morphometry provides highly objective symmetry indices, it does not directly reflect subjective aesthetic perception. Future studies integrating patient reported outcomes, expert aesthetic ratings, and quantitative shape analysis may offer a more comprehensive assessment of postoperative quality.

## 5 Conclusions

In summary, this longitudinal 3D investigation demonstrated that postoperative morphological changes after primary cheiloplasty occur in a region-specific manner. Symmetry of the lateral white lip exhibited a gradual improvement during the first postoperative year, whereas asymmetry at the columellar base and nasal floor persisted. These findings emphasize the importance of considering differential remodeling capacity across anatomical subregions when designing the immediate postoperative configuration, which may contribute to a refinement of primary surgical strategies aimed at achieving more stable and aesthetically favorable long-term outcomes in patients with cleft lip.

## Data Availability

The raw data supporting the conclusions of this article will be made available by the authors, without undue reservation.
